# Open Radiocarpal Fracture Dislocation with Neurological Deficit Treated with Standalone External Fixation and Kirshner-Wires: Evaluation of Functional and Radiological Outcomes in a 4-Year Follow-Up: A Rare Case Report

**DOI:** 10.3390/reports9010057

**Published:** 2026-02-10

**Authors:** Constantinos Chaniotakis, Christos Koutserimpas, Petros Kapsetakis, Alexandros Tsioupros, Kalliopi Alpantaki

**Affiliations:** 1 Department of Orthopaedics and Traumatology, “Venizeleion” General Hospital of Heraklion, 44 Leoforos Knossou, 71409 Heraklion, Greece; petkas79@hotmail.com (P.K.); alexandros-tsioupros@hotmail.com (A.T.); apopaki@yahoo.gr (K.A.); 2 School of Health Rehabilitation Sciences, University of Patras, 26504 Patras, Greece; chrisku91@hotmail.com

**Keywords:** radiocarpal dislocation, open fracture, external fixation, wrist injuries, Health-Related Quality of Life

## Abstract

**Background and Clinical Significance**: Radiocarpal fracture dislocations (RCFDs) are rare injuries of the wrist, while open RCFDs represent a small subgroup of these injuries. Limited data exists regarding the optimal method for their management. Our study’s objective is to present a rare case of an open (Gustilo–Anderson type II) dorsal radiocarpal dislocation in combination with fracture of the radial and ulnar styloid and neurologic deficits (superficial radial, median and ulnar nerve), which was treated with external fixation and Kirshner wire pinning. External fixation and Kirshner wire pinning could be a viable surgical option for complicated open RCFD. **Case Presentation**: Adequate reduction and ligamentotaxis using an external fixation were achieved, while the radial styloid fracture and the distal radioulnar joint (DRJ) were stabilized with Kirshner wires. Postoperative radiographs and clinical evaluation confirmed satisfactory reduction in the right wrist, without signs of intercarpal instability. Total nerve recovery was observed 6 months postoperatively and the patient was able to return to his previous occupation. At the final follow-up (4 years), the Visual Analogue Scale score was 1/10 and the Quick Dash score was 11/100 with good range of motion (flexion: 0–75°, extension: 0–70°, pronation: 0–80°, supination: 0–80°) of the affected wrist, although progressive wrist arthritis and ulnar migration was seen in the plain X-rays. **Conclusions**: Surgical treatment of RCFDs is required for complex or unstable fractures/dislocations to avoid possible complications, such as intercarpal instability.

## 1. Introduction and Clinical Significance

Radiocarpal fracture dislocations (RCFDs) are extremely rare injuries of the upper extremity, usually occurring after high-energy traumatic events [1,2]. Furthermore, open RCFDs comprise a small subgroup with only a few studies describing this type of injury [3,4]. These injuries’ mechanism is related to hyperextension, ulnar deviation and hyperpronation. More frequently than volar dislocations, dorsal RCFDs account for 60% of all cases [5]. RCFDs differ from lunate and perilunate dislocations, since the intercarpal connection is preserved [6]. In cases of open fractures-dislocations, adequate debridement, antibiotic prophylaxis, and release of the neurovascular structures should be performed within hours of trauma [7].

Dumontier’s and Moneim’s classifications are the most commonly used to classify these injuries, and they are based on the presence or absence of an associated radius styloid fracture or intercarpal instability respectively (Table 1) [8,9,10].

Jupiter et al. mentioned that neurologic dysfunction may be present in patients with RCFDs [11]. Moreover, several complications, such as intercarpal instability, carpal non-union, later arthrosis, ulna translation and loss of radiocarpal stability have also been reported. The median nerve is more frequently compressed or contused than the ulnar nerve, especially in open injuries [11]. Due to the rarity of these injuries, there is no consensus on the optimal management [12]. Closed reduction and cast immobilization have limited indications and surgery is often required, especially for complex or unstable injuries [6,13,14].

In this article, we describe a rare case of a patient who presented with an open (Gustilo–Anderson type II) dorsal radiocarpal dislocation with an ulnar opening, associated with a fracture of the radial and ulnar styloid with severe deformity and median, superficial radial, and ulnar nerve deficits. The surgical management of this injury, along with the functional and radiological outcomes after a 4-year follow-up, is reported.

## 2. Case Presentation

**Figure 1:**
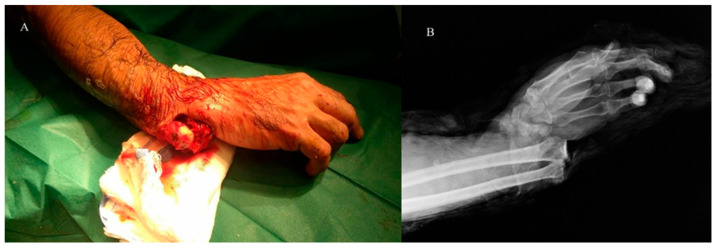
(**A**): The open injury upon presentation. (**B**): Anteroposterior X-ray view of the wrist. Dorsal radiocarpal dislocation of the right wrist (pre-reduction).

A 43-year-old male arrived in our emergency department following a motorcycle accident. Upon presentation, he was oriented, hemodynamically stable (blood pressure: 135/87 mmHg, heart rate: 88 beats/min) and afebrile. Clinical and imaging evaluation (plain radiographs) revealed an open (Gustilo type II) dorsal radiocarpal dislocation of the right wrist with exposed distal radial joint surface, with a fracture of the radial and ulnar styloid (Dumontier II) (Figure 1).

Neurological examination was inconclusive due to severe pain and distress. Both radial and ulnar pulses were palpable on the right wrist. Concomitant injuries included a contralateral closed diaphyseal femoral fracture.

Administration of intravenous antibiotics (cefoxitin 1 g and amikacin 1 g), irrigation and debridement of the wrist were the first steps of treatment. A posterior above-knee back slab was applied to immobilize the closed femoral fracture and urgent manipulation to reduce the radiocarpal dislocation was performed under sedation using longitudinal traction. However, the dislocation was irreducible due to soft tissue interposition.

The patient was immediately taken to the operating room for further surgical treatment under general anesthesia. Exploration of the open trauma of the wrist did not reveal any tendon or neurovascular injuries. The radiocarpal joint was inspected through the site of the disruption. The joint was thoroughly irrigated and debrided of any loose cartilage or bone fragments. Under fluoroscopic guidance, adequate reduction and ligamentotaxis were achieved, followed by application of radiocarpal external fixation. Τwo pins were placed in the radial diaphysis and two pins in the diaphysis of the second metacarpal. Both the radial styloid fracture and the distal radioulnar joint (DRJ) were stabilized with Kirshner wires. The femoral fracture was also treated with antegrade intramedullary nailing.

Postoperative radiographs confirmed a satisfactory reduction in the right wrist. No signs of intercarpal instability were found (Moneim I). The neurological status was re-evaluated postoperatively, and superficial radial, median and ulnar nerve palsy with motor and sensory deficits was found. After 5 days of hospitalization, the patient was discharged.

**Figure 2:**
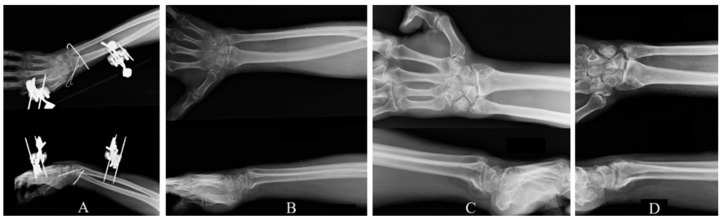
AP and Lateral X-ray views of right wrist at (**A**): 2-month, (**B**): 12-month, (**C**): 2-year, (**D**): 4-year follow-ups.

The Kirshner wires and the external fixation were removed two months postoperatively and a dynamic splint was placed, while the patient started rehabilitation with passive and active wrist motions. Nerve conduction studies six weeks postoperatively confirmed the neurological deficits, revealing severe damage to the ulnar, median and superficial radial nerve. Clinical and radiological evaluation of the right wrist was performed at distinct follow-up intervals at 1, 3, 6, 12 months and 2, 3, 4 years postoperatively (Figure 2).

The assessment comprised clinical examination (neurological status and range of motion) and implementation of well-established patient-reported outcomes measures, including the Visual Analogue Scale (VAS) and Quick Dash score for evaluation of pain and disability, as well as the Short-Form (SF-36) Medical Health Survey Questionnaire for Health-Related Quality of Life (HRQoL) analysis. Total nerve recovery was observed 6 months postoperatively and the patient was able to return to his previous occupation (manual labour) and hobbies. A continuous improvement in all indices of SF-36 over the entire follow-up period was noticed, with a plateau at 2 years (Table 2).

Table 3 presents VAS and Quick Dash scores and the range of motion of the right wrist at each follow-up period.

**Figure 3:**
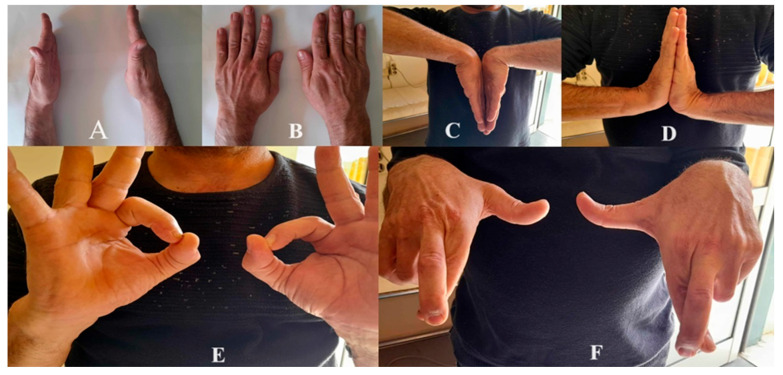
Range of motion and neurological examination of the right wrist, compared to the opposite healthy side, at the final follow-up. (**A**): Starting potion. (**B**): Pronation. (**C**): Flexion. (**D**): Extension. (**E**): “OK” sign (test for anterior interosseous nerve). (**F**): Crossing index and long fingers (test for ulnar nerve motor function).

At the final follow-up (4 years), the VAS score was 1/10, the Quick Dash score was 11/100, and there right wrist had a good range of motion (flexion: 0–75°, extension: 0–70°, pronation: 0–80°, supination: 0–80°) (when compared to the opposite healthy side), without neurological deficits (Figure 3).

Neurological function was additionally evaluated using the Medical Research Council (MRC) scale for both muscle strength and sensory function. With regard to muscle strength, a progressive improvement was documented, with an MRC score of 3/5 after 3 months postoperatively and full recovery (5/5) at 6 months. Sensory function demonstrated a similar pattern of recovery, improving from 1/2 after 3 months to 2/2 at 6 months. Regarding the Two-Point Discrimination Test, a discrimination threshold of 6 mm was recorded after the 3-month follow-up; prior to this time point, complete palsy had been observed on neurological sensory examination. Sensory discrimination values returned to normal at the 6-month follow-up.

However, progressive wrist arthritis and ulnar migration were observed in the plain X-rays.

## 3. Discussion

RCFDs are rare and complex injuries, accounting for just 0.2 to 2.7% of all wrist injuries [15]. The present case belongs to a scarce subgroup of open RCFDs with concomitant neurological deficit of three nerves (ulnar, median and superficial radial nerve). Several studies involving a limited number of patients, as well as collections of individual case reports, are used to support the current understanding of this injury and its management. Radial or ulnar styloid fractures are frequently seen. More than half of the scaphoid fossa is usually involved in the attachment of the radioscaphocapitate, radioscaphoid, and long radiolunate ligaments to radial styloid fragments. Strong attachments of the short radiolunate and radioscaphocapitate ligaments can also result in radial rim avulsion fractures, along the articular margin of the distal aspect of the radius [16].

Non-operative treatment of these injuries with casting has poor outcomes, resulting in late instability typically necessitating surgery, and it is only indicated in a small percentage of cases with purely ligamentous injuries when the radiocarpal joint is found stable after the reduction [17]. There have been many surgical procedures proposed, including external fixation, ligamentous repair, screw fixation of the styloid fragment, and Kirshner wire fixation. Identifying and treating intercarpal injuries, reducing dislocation, and repairing all ligamentous avulsions are the cornerstones of treatment, but there is no consensus regarding optimal surgical approach [18].

A major challenge of the successful management of this injury is providing a stable and congruently reduced joint [19]. Bamal et al. mentioned that the key steps in achieving and maintaining reduction are to address the fixation of the radial styloid, the volar teardrop fragment of the distal radius and the short radiolunate ligament (SRL) [6]. De Villeneuve Bargemon et al. highlighted the three major components (bone component, ligament component and associated intercarpal lesions) in the treatment of these complex injuries and proposed a specific management plan [14]. The predominant complication is stiffness, resulting in a 30–40% decrease in the range of motion in flexion/extension. Mugdal et al. reported 12 patients who presented with RCFDs. The mean wrist motion of these individuals, at a mean follow-up of 36 months, was 59° of flexion, 53° of extension, 74° of pronation and 82° of supination [20]. Table 4 presents other cases that mentioned the range of motion of the affected wrist at final follow-up.

Open lesions, pure dislocations (type I in Dumontier classification), additional nerve injury, intercarpal lesions and injuries on the same limb indicate poor prognosis [7]. The reported case was an open injury with severe deformity and substantial nerve damage. However, the patient had a satisfactory functional outcome at the 4-year follow-up.

In cases with irreducible dislocations, the main block of closed reduction is usually a volar distal radial fragment with attached ligamentous structures [22]. Open exploration and appropriate surgical management can lead to good functional outcomes [22]. Jardin et al. described a case of open volar radiocarpal dislocation with complete rupture of the dorsal capsule–ligament complex and tendons [1]. The authors proposed that anatomically repairing the capsule–ligament complex via the dorsal and anterior approach, along with tendon and temporary bone fixation using Kirshner wires or external fixation, can minimize postoperative complications [1]. We believe that in our case, the failure of initial closed reduction in the emergency department was mainly due to the soft tissue interposition. For this reason, we hastened surgical intervention in order to facilitate reduction in the dislocation and decompression of the neural and vascular structures.

Yuan et al. reported that open reduction, internal fixation and repair of the ligaments offer improved long-term functional outcomes when compared to acute partial or full wrist arthrodesis [23]. Spiry et al. presented a study of 41 patients with RCFDs [24]. In cases of pure radiocarpal dislocation, the authors performed reduction and stabilization by a dorsal approach without volar capsule and ligament suturing. Patients with concomitant radial fractures were treated with closed anatomical reduction and radiostyloid pinning. The authors noted that effective reduction and radiocarpal stability, as well as the absence of radial and intercarpal marginal fractures, resulted in good functional outcomes and no osteoarthritis [24]. Cornu et al. reported favourable results without ulnar translation or radiocarpal subluxation in a case series of 14 patients with RCFDs (Dumontier type I), without performing any ligament reconstruction, concluding that internal fixation combined with wrist rigid immobilization is sufficient to facilitate ligament healing [25]. Potter et al. proposed distraction plating as a safe and effective technique for treating selected distal radius fracture–dislocations [21].

Closed reduction without ligament repair or internal fixation, as in our case, is supported by Moneim et al. in Type I dislocation [8]. According to Moneim et al., less trauma occurs in Type I dislocation, and the ligamentous disruption occurs as a single unit between the distal radius and the carpus; thus, closed reduction is a treatment option for this type of dislocation [8]. When closed reduction is not successful because of the interposition of bone fragments in the joint, or if anatomical reduction in the radial styloid is necessary, they advise open reduction. Cross-pinning of the radiocarpal joint can avoid subluxation in the future if the reduction is unstable [8]. Apergis et al. recommend the application of external fixation because the wrist can be kept gross in alignment and mild destruction facilitating soft tissue healing in proper tension [12]. Additionally, our treatment strategy is supported by Dumontier et al. [9]. The volar radiocarpal ligaments are most often intact and remain attached to the radial fragment (styloid process) in type II injuries, according to the authors’ classification. The most crucial stage in treating these patients is thought to be fixing the fracture fragment. They also advocate using a cast or an external fixation for at least six weeks [9]. In our case, anatomic reduction in the radial fragment was not accomplished. Dumontier et al. also mention the difficulty of closed anatomic reduction in the radial fragment, and they suggest open reduction and fixation in these cases [9].

Nyquist and Stern reported the largest series (ten patients) of open RCFDs, treated with wound debridement, open reduction and additional casting supplemented with internal or external fixation, reporting rather poor results in terms of pain and stiffness [4]. In seven cases, the median and/or ulnar nerve was severely contused and required immediate decompression. All patients had some sensory loss during follow-up. The authors expressed their doubt that nerve dysfunction in open injuries is caused by nerve compression (because the dislocations were open, and they routinely decompressed all the patients). They believe that in open RCFDs, the amount of trauma energy is even greater than that of closed injuries and results in nerve damage [4]. These injuries are associated with high-energy trauma, and this may have a long-term negative impact on the wrist joint, even after adequate reduction and stabilization. It is important to note that in this case, progressive wrist arthritis and ulnar migration were observed in the plain X-rays. The uniqueness of the present case lies in the fact that three nerves (median, ulnar and superficial radial nerve) were damaged. Remarkably, our patient achieved complete nerve recovery six months postoperatively. Given that all nerves were found intact, the anticipated resolution of nerve contusion and traction neuropraxia following reduction and stabilization was confirmed.

## 4. Conclusions

There is a scarcity of data in the recent literature investigating the gold standard management of RCFD. There is no consensus for a specific therapeutic choice for each type of RCFD, due to the rarity of this injury. However, external fixation and Kirshner wire pinning could be a surgical treatment of open RCFD in many cases. Longer-term and larger-scale research is required to assess the appropriate treatment option and functional outcomes.

## Figures and Tables

**Table 1:** Dumontier’s and Moneim’s radiological classification systems.

Dumontier Classification [9]	Moneim Classification [8]
Type I: Pure radiocarpal dislocation or with only radial styloid fracture	Type I: Radiocarpal fracture-dislocation without associated intercarpal dissociation.
Type II: Radiocarpal dislocation with a radial styloid fracture that involved more than one-third of the width of the scaphoid fossa.	Type II: Radiocarpal fracture-dislocation with an associated intercarpal dissociation.

**Table 2:** Indices of SF-36 at each follow-up period.

Indices of SF-36	Postoperative Follow-Up						
	1 Month	3 Months	6 Months	12 Months	2 Years	3 Years	4 Years
PF (%)	35	57	69	79	96	96	100
RP (%)	37	49	62	77	96	96	100
BP (%)	31	61	72	84	98	98	100
GH (%)	33	55	70	82	98	98	100
V (%)	31	49	68	81	98	98	100
SF (%)	28	45	64	82	96	96	100
RE (%)	29	47	66	83	98	98	100
MH (%)	34	50	69	81	98	98	100

**Table 3:** VAS, Quick Dash scores and range of motion of the right wrist at each follow-up period. We did not assess the range of motion of the right wrist until the first 3 months of the follow-up due to the neurological deficit.

Follow-Up	Scores					
	VAS	Quick Dash	Range of Motion of the Right Wrist			
			Flexion	Extension	Pronation	Supination
1 month	4/10	61/100	-	-	-	-
3 months	3/10	45/100	-	-	-	-
6 months	1/10	34/100	0–45°	0–49°	0–45°	0–47°
12 months	1/10	17/100	0–67°	0–62°	0–71°	0–72°
2 years	1/10	11/100	0–75°	0–70°	0–80°	0–80°
3 years	1/10	11/100	0–75°	0–70°	0–80°	0–80°
4 years	1/10	11/100	0–75°	0–70°	0–80°	0–80°

**Table 4:** Range of motion of affected wrist after surgical treatment at final follow-up.

Authors	Range of Motion				Final Follow-Up
	Flexion (°)	Extension (°)	Pronation (°)	Supination (°)	
Woon CY et al. [2]	21	11	80	76	1 year
Jardin E et al. [1]	65	20	-	-	18 months
Potter MQ et al. [21]	65	70	Full	Full	1 year
Yang P et al. [22]	70	50	Full	Full	18 months
Our case	75	70	Full	Full	4 years

## Data Availability

The original data presented in the study are included in the article; further inquiries can be directed to the corresponding author.

## Abbreviations

The following abbreviations are used in this manuscript:

RCFDs	Radiocarpal Fracture Dislocations
CT	Computed Tomography
DRJ	Distal Radioulnar Joint
VAS	Visual Analogue Scale
SF-36	Short-Form-36
HRQoL	Health-Related Quality of Life
SRL	Short Radiolunate Ligament
AP	Anteroposterior
PF	Physical Functioning
RP	Role-Physical
BP	Bodily Pain
GH	General Health
V	Vitality
SF	Social Function
RE	Role-Emotional
MH	Mental Health
